# The Influence of Background Music on Learning in the Light of Different Theoretical Perspectives and the Role of Working Memory Capacity

**DOI:** 10.3389/fpsyg.2017.01902

**Published:** 2017-10-31

**Authors:** Janina A. M. Lehmann, Tina Seufert

**Affiliations:** Department of Learning and Instruction, Ulm University, Ulm, Germany

**Keywords:** learning with background music, arousal-mood-hypothesis, Mozart effect, seductive detail effect, working memory capacity, aptitude-treatment interaction

## Abstract

This study investigates how background music influences learning with respect to three different theoretical approaches. Both the Mozart effect as well as the arousal-mood-hypothesis indicate that background music can potentially benefit learning outcomes. While the Mozart effect assumes a direct influence of background music on cognitive abilities, the arousal-mood-hypothesis assumes a mediation effect over arousal and mood. However, the seductive detail effect indicates that seductive details such as background music worsen learning. Moreover, as working memory capacity has a crucial influence on learning with seductive details, we also included the learner’s working memory capacity as a factor in our study. We tested 81 college students using a between-subject design with half of the sample listening to two pop songs while learning a visual text and the other half learning in silence. We included working memory capacity in the design as a continuous organism variable. Arousal and mood scores before and after learning were collected as potential mediating variables. To measure learning outcomes we tested recall and comprehension. We did not find a mediation effect between background music and arousal or mood on learning outcomes. In addition, for recall performance there were no main effects of background music or working memory capacity, nor an interaction effect of these factors. However, when considering comprehension we did find an interaction between background music and working memory capacity: the higher the learners’ working memory capacity, the better they learned with background music. This is in line with the seductive detail assumption.

## Introduction and Theoretical Background

Music has become much more readily available to the public in the past decades. One influencing factor was the increasing availability of music: whilst in the past one was in need of CDs or tapes and an according player, nowadays music can be played digitally on many different devices such as computers, mobile phones or iPods. Furthermore, the choice of available songs is almost endless due to music portals. This makes it possible to select suitable songs for different situations, such as relaxing songs for a cozy evening or activating songs before going out. Due to these advances in music technology, learning with background music has received more and more attention over the last decade (e.g., Schwartz et al., 2017).

For some situations it seems intuitive to think that music would help to enhance our experience – but how do music and learning fit together? At present the effects of background music while learning and the mechanisms behind this are unclear. On the one side, music seems to have a positive (Mozart effect; Rauscher et al., 1993) and stimulating effect (arousal-mood-hypothesis; Husain et al., 2002), which could improve learning. On the other side, background music could lead to an additional burden on working memory (seductive detail effect; e.g., Rey, 2012), thus hindering learning. To be able to simultaneously deal with the learning material and the background music, the learner’s working memory capacity is a crucial factor to consider.

### Background Music

In this study we define background music as music that plays in the background while studying, i.e., when reading a text. Learners are intended to listen to this music but there is no relation between the music itself and the main task, namely learning the text.

Results of studies investigating the relationship between background music and learning outcomes are varied. While some studies found no effect of background music (e.g., Moreno and Mayer, 2000; Jäncke and Sandmann, 2010) others found that it negatively impacted learning outcomes [e.g., Furnham and Bradley, 1997; Randsell and Gilroy, 2001; Hallam et al., 2002 (study 2)]. Further studies report that it has a positive impact [e.g., Hallam et al., 2002 (study 1); de Groot, 2006], especially on students with learning disabilities (Savan, 1999) or poor spelling skills (Scheree et al., 2000).

Thompson et al. (2011) gave a first hint as to why previous results were so mixed. They revealed that music characteristics like tempo and intensity have an influence on learning outcomes: only soft fast music had a positive influence, whilst loud fast as well as soft slow or loud slow music hindered learning. In addition, instrumental music disturbs learners less than music with lyrics (Perham and Currie, 2014). As each study used their own music and did not control for the characteristics of their music choice, this is one possible explanation for the heterogeneous study results mentioned above. Moreover, it seems plausible that learner’s characteristics such as their musical expertise (Wallace, 1994) or their familiarity with the presented music could also impact their learning.

Importantly, it is not the characteristics of a song *per se*, but their effects on the learner which influence learning outcomes. These effects on the learner have been explained by different theoretical approaches. These can be grouped into approaches positing either a potentially positive or negative influence on learning outcomes.

The first theoretical perspective explains why background music could positively influence learning and cognitive abilities. Probably the most well-known approach in this field is the so-called Mozart effect (Rauscher et al., 1993). In this study, before completing a task that measured spatial abilities, some participants listened to a Mozart sonata, while others did not listen to any music. Participants in the Mozart condition outperformed the other group. The authors found a direct, positive influence of listening to Mozart sonatas on spatial abilities. They explain these better test results though priming effects. Even though in the experiment the exposition to music took place in advance of the task, the results are transferrable to listening to music while learning. Priming effects should be even stronger during the exposition to the stimulus and decay over time (e.g., Foss, 1982).

This priming explanation, however, was criticized by Husain et al. (2002). They formulated the arousal-mood-hypothesis. It states, that listening to background music does not have a direct influence on cognitive abilities, but affects it through the mediators of arousal and mood. The prerequisite for this assumed mediation is that background music has an impact on arousal and mood, which in turn impact learning outcomes. Moreover, the authors go one step further and postulate that this mediation effect should not only influence spatial abilities, but also cognitive performance.

When considering arousal, Husain et al. (2002) follow Sloboda and Juslin’s (2001) definition, that arousal describes physical activation. The influence of listening to background music on arousal (for an overview, see Pelletier, 2004) is well-established: Music can increase or decrease arousal, mostly influenced by the tempo of a song (Husain et al., 2002). In addition, there is broad evidence of the impact of arousal on learning (e.g., Kleinsmith and Kaplan, 1963; Eysenck, 1976; Heuer and Reisberg, 2014). The Yerkes–Dodson law (Yerkes and Dodson, 1908) describes optimal arousal in a learning situation following an inverted U-shaped pattern. While learners with little arousal are not engaged enough to really invest in the learning process, too much arousal can cause distractive feelings like anxiety. Thus, a medium level of arousal is optimal for learning. In conclusion, a mediation effect of background music over arousal on learning seems probable, as there seems to be an influence of background music on arousal as well as an impact of arousal on learning.

When considering mood, the arousal-mood-hypothesis defines mood as referring to emotions (Sloboda and Juslin, 2001). Several studies have found background music to influence mood (e.g., Juslin and O’Neill, 2001; Sloboda and Juslin, 2001; Schmidt and Trainor, 2010). Background music leads to different emotions dependent on whether they are composed in a major or minor mode (Husain et al., 2002). Moreover, several theoretical approaches and studies state that mood influences learning (Ilsen, 1984; Pekrun, 2006; Goetz and Hall, 2013; Heuer and Reisberg, 2014; Pekrun et al., 2017). In general, positive mood is associated with better learning outcomes (Isen, 2002) while negative mood or boredom hinders learning (O’Hanlon, 1981; Pekrun, 2006). Based on this, a mediation effect of mood also seems plausible.

To conclude, Husain et al. (2002) state that besides these two mediation effects (mood and arousal mediating the influence of background music on learning) and in contrast to the Mozart effect, music does not directly influence learning. The authors underpinned this statement by referring to a study by Nantais and Schellenberg (1999). In this study participants listened to a Mozart sonata and to a short story and completed a spatial task after each. Participants were also asked if they liked the sonata or the story better. In general, participants performed better after listening to the stimulus (sonata or story) they preferred. Thus, Husain et al. (2002) reasoned that better cognitive performance when listening to background music is due to the exposure to a pleasant stimulus.

In sum, both the Mozart effect and the arousal-mood-hypothesis state that listening to background music can foster learning, while the arousal-mood-hypothesis also takes characteristics of the melody into account. A piece of music needs to be in the right tempo and mode to be able to evoke the appropriate arousal and mood in the learner. When investigating arousal and mood evocation, it is not enough to simply measure arousal and mood after learning, but measurements need to be taken before and after learning. Only in this way is it possible to calculate the change in arousal and mood during the learning phase.

Another completely contradictory theoretical perspective describes why background music can also have a negative impact on learning. When learning with background music, the learners have to divide their attention between the learning task and the music. Thus, they have to invest cognitive resources to process the background music in addition to the learning task, as auditive information always gets processed first (Salamé and Baddeley, 1989) and cannot be ignored (Mayer, 2001). Background music is not related to the task, but can attract the learner’s attention and therefore can be defined as a seductive detail (Rey, 2012). Such information distracts the learner from the main task, i.e., the learning task, and therefore hinders learning. Hence, it is not surprising that a meta-analysis of the influence of background music that involved many types of music (including different tempi and modes) (Kämpfe et al., 2010) revealed an overall negative impact on learning. Music becomes an unnecessary burden on working memory, which is a crucial point when regarding the limitations of working memory capacity (Miller, 1994; Cowan, 2001).

### Working Memory Capacity

The importance of working memory and its capacity in a learning situation is due to the fact that all information within a learning situation (including learning material, learning task, and context factors) needs to be processed within working memory. There is an ongoing debate about the structure of working memory. Baddeley (1986) and Cowan (1999) published probably the two most prominent working memory models. As the experimental group in this study has to deal with visual (reading a text) as well as auditive information (listening to background music) we will especially focus on how this information gets processed according to Baddeley’s (1986) and Cowan’s (1999) models.

Baddeley (1986) assumes working memory to be a system with a hierarchical structure: the central executive controls the two subsystems which are phonological loop and visuospatial sketchpad. He postulates that working memory is separated to long-term memory, even though long-term memory can have an influence on processes within working memory. For example, prior knowledge activated in long-term memory can facilitate the processing and integration of new information in working memory. Due to different independent subsystems, which work in parallel and all involve their own independent capacity, it is easier to process information of different modalities. A visual text is processed with the phonological loop after being recoded through subvocal processes. Background music is phonological information as well as it is presented auditory, and thus might overload the phonological loop. However, there is evidence that musical information gets processed in a slightly different way to verbal auditive information (Salamé and Baddeley, 1989).

Different authors assume an additional, subsystem to be responsible for processing background music, which is partly independent from the phonological loop (Deutsch, 1970; Rowe et al., 1974; Paivio et al., 1975; Rowe, 2013). Referring to this, there is more capacity available while processing music in addition to a visual text as two different subsystems are utilized, compared to the processing of auditive text in addition to a visual text processed in the same subsystem. As such, background music would still interfere with reading, but not as severely as, for example, when verbal auditive information is processed by the same subsystem.

Another approach to working memory was put forward by Cowan (1999) who proposed the embedded-processes model. Working memory in this model is the activated part of long-term memory, without differentiating between the processing of different modalities. Cowan argues, that the similarity of information has an influence on how much information can be processed simultaneously: the less similar the content and modality of the information is, the easier it is to process them simultaneously. Concerning instrumental background music and reading a text at the same time, this would mean that instrumental music would be less disruptive compared to music with lyrics or a classical auditive text because of the added verbal aspect. However, processing background music still relies on the same cognitive capacity, thus, hindering learning.

Independent of which model describes working memory better, they both assert that listening to background music while learning requires additional cognitive capacity that could otherwise be invested into the learning process. This is especially important, as working memory capacity is limited.

Working memory capacity can be defined as the number of separate concepts that can be dealt with at the same time in working memory (Cowan, 2012). Cowan (2001) states that 3–4 chunks of information can be stored and manipulated at the same time. A wide variety of studies show an advantage in learning situations for learners with a higher working memory capacity [e.g., Daneman and Carpenter, 1983; King and Just, 1991; Whitney et al., 1991; Rosen and Engle, 1998 (Experiment 1); Alloy and Alloy, 2010]: the more information an individual can deal with simultaneously, the more efficient the learning process. However, listening to background music reduces the available memory capacity for the learning process. How then do background music and working memory capacity interact?

### Interaction between Background Music and Working Memory Capacity on Learning

Salamé and Baddeley (1989) postulate firstly, that it is impossible not to process auditive information and secondly, that auditive information is always processed first. Thus, only if working memory capacity is high enough do learners have sufficient capacity to invest in the learning task after processing the auditive information. In this case, appropriate background music could be of benefit to learners by influencing their mood and arousal level to an optimal state, thereby fostering the learning process. However, even for those learners melodies should be chosen that only pose a small burden on working memory. Comparing instrumental music with songs with lyrics, it seems plausible that when lyrics are present they would need to be additionally processed. According to Baddeley’s (1986) model, these lyrics are auditive texts that burden the phonological loop, leading to a larger decrease in learning performance compared to an instrumental song. The same is true for Cowan’s (1999) model, where the lyrics are too similar to the visual text and therefore lead to interferences during learning.

Therefore, when attempting to foster learning for high-capacity learners by improving mood and arousal, one should use a music without lyrics. In this case learners may be able to process the learning material as well as the song. Therefore, sufficient working memory capacity may compensate for the additional cognitive burden, so that the potential positive effect of the music may benefit the learner. This is comparable to the ability-as-compensator effect (Mayer and Sims, 1994), where a learner’s ability (in this study: sufficient working memory capacity), is required to deal with a specific element of the instructional design (in this study: Background music).

When learners with low working memory capacity have to process background music there is not enough capacity left to invest in the learning task. Even if the learners were in a perfect learning condition concerning arousal and mood, they would not be able to learn as they simply would not be able to process the information in the learning material in addition to the music.

To our knowledge, there is no empirical evidence of the interaction between background music and working memory capacity on learning outcomes which could support these theoretical assumptions. As we defined background music as a seductive detail, we argue that research on other seductive details in interaction with working memory capacity might be transferrable. Sanchez and Wiley (2006) found, that learners with low working memory capacity were hindered in their learning if learning materials included seductive pictures in addition to the text. Interestingly, learners with higher working memory capacity were not affected by these pictures, however, their performance did not increased either. As the pictures used in Sanchez and Wiley’s (2006) experiment were normed to not influence arousal or mood as our experiment does, this result is not contradictory to our assumptions. A study by Fenesi et al. (2016) found similar results: Learners with low working memory capacity perform worse when presented with irrelevant pictures in addition to learning material.

The cut-off between a working memory capacity that is “too small” and “high enough” depends on the characteristics of the learning material. Highly complex or poorly designed learning tasks burden working memory capacity more than content which is less complex or better designed (Sweller, 2010; Sweller et al., 2011). This indicated that background music should only be considered when the learning material itself is not too demanding. A similar effect is was found in a study by Park et al. (2011) where pictures were used as a seductive detail. The researchers varied the complexity of the main task and found that pictures hindered learning less when the main task was not very demanding, whereas the seductive details effect was revealed with highly demanding tasks.

### Learning Outcomes

Besides the complexity of the learning material, the level of learning outcomes could also play an important role. So far, we have discussed learning outcomes in general. However, one can differentiate between different levels of learning outcomes, like recall or comprehension (e.g., Bloom, 1956). For exams it is typically necessary to remember and understand the learning content. Thus, the post-test of this study differentiates between both of these learning outcomes. To our knowledge no studies as yet differentiate between the influence of background music on recall and comprehension, so we can only establish assumptions on a theoretical basis and turn to results of comparable studies for comparisons. As cited above, in a study by Park et al. (2011) the seductive detail effect depended on task difficulty with easy tasks not affected by seductive details. Transferring these results to learning with background music and to different levels of learning outcomes, i.e., recall and comprehension, one would expect background music to influence comprehension outcomes but not recall. Easier recall tasks are a smaller burden in working memory so that a learner may be able to process background music simultaneously. In addition, working memory capacity does not play an important role, as the learner does not need a high capacity. This is also why also the interaction between both factors should not influence recall performance.

However, comprehension tasks are more demanding and are bigger cognitive burdens. In this case, background music should affect comprehension outcomes, as well as working memory capacity. Moreover, we should witness an interaction between both factors in the way described above.

### Research Questions and Hypothesis

To sum up, the influence of background music on learning is not clear: while the Mozart effect (Rauscher et al., 1993) implies a direct, positive effect, the arousal-mood-hypothesis (Husain et al., 2002) postulates a mediation effect over arousal and mood. Furthermore, the seductive detail effect indicates that background music has a direct negative effect on learning. In addition, the level of learning outcomes could also play an important role. On this basis, we pose the following research questions: Does listening to background music influence learning directly or is this association mediated by arousal or mood? And which role does the learner’s working memory capacity have and how does it interact with background music?

All three theoretical assumptions (Mozart effect, arousal-mood-hypothesis and seductive detail effect) have theoretical and empirical justifications. As we are the first to compare all three of these, we formulate the following in parts competing hypotheses: Background music does not influence recall (H1.1), but comprehension (H1.2):

H1.2a: Due to the Mozart effect, comprehension will be influenced positively and directly by background music.
H1.2b: Due to the arousal-mood-hypothesis, we hypothesis that arousal and mood will be related to music and learning outcomes. As we chose music that was intended to induce positive mood and learning enhancing arousal, we expect background music to influence mood positively, thus fostering comprehension. Secondly, we expect that background music to have a positive impact on arousal, with arousal improving comprehension.
H1.2c: On the basis of the seductive detail effect, we hypothesize that there will be a direct negative influence of background music on comprehension.

Several studies cited above found better learning outcomes for learners with higher working memory capacity. As we think that a higher working memory capacity is only necessary for more demanding tasks, we hypothesize that there will be no main effect of working memory capacity on (H2.1) recall but on (H2.2) comprehension, with better comprehension scores recorded for learners with higher working memory capacity.

There is a lack of research investigating the interaction between listening to background music and working memory capacity. Theoretically, we assume that learners with low working memory capacity will be overburdened by processing both the learning material and the background music. Nevertheless, learners with sufficiently high working memory capacity could benefit from the potential positive effect of background music which compensates for the additional cognitive burden (see Mayer, 2001). However, this should only be relevant for comprehension tasks which are highly demanding. Based on these theoretical assumptions and the results of transferrable studies, we hypothesize that there will be (H3.1) no interaction effect between background music and working memory capacity on recall. However, we hypothesis that (H3.2) this interaction effect will be present in the case of comprehension. More specifically, we hypothesise that there will be (H3.2a) better comprehension outcomes for learners with low working memory scores while not listening to background music. Learners with high working memory capacity, (H3.2b) will have better comprehension outcomes when listening to background music while learning.

## Materials and Methods

### Subjects and Design

Data was collected from 86 university students aged between 16 and 50 years (*M*_age_ = 21.37, *SD*_age_ = 4.19), including 71 (82.6%) females. Due to their very poor test performance, five participants were defined as outliers (e.g., Barnett and Lewis, 1994). We compared all post-test scores to the predefined criteria of 20% of the possible post-test score. As these five participants reached less than 15% of the post-test score, we assume that they were not engaged enough in the learning process and we excluded their data. Hence, data from 81 participants (*M*_age_ = 21.46, *SD*_age_ = 4.30, 81.5% females) were included in further analysis.

Participants were randomly assigned to one experimental group (between-subject factor: Background music – present or absent). Working memory capacity was included in the design as an organism variable, also considered as an independent variable. As dependent variables, we measured recall and comprehension as indicators for learning performance. In addition, we measured mood and arousal as potential mediating variables. Moreover, we considered prior knowledge, musical experience, age and gender as potential covariates.

### Materials and Measures

All materials besides the background music and the instruction to learn were in paper-pencil form. Due to our materials, there was no ethics approval needed for this study.

The *learning material* consisted of a visual text about time and date differences on earth that was 1070 words long. It was adapted from a study of Schnotz and Bannert (1999). The adapted version of the learning materials has successfully been used in another study by Lehmann et al. (2016). The text includes information about the concept of time and time zones as well as a table that shows exemplary time differences between different cities around the world. Learning time was limited to 7 min and 30 s. To accompany the text a test to measure *prior knowledge* was created. It consisted of six open-ended questions (e.g., “What are time zones?”). Answers were compared to predefined solutions. *Learning outcomes* were measured using five open-ended recall questions (e.g., “According to which principle were the time zones classified?”) and five open-ended comprehension questions (e.g., “What time is it in Frankfurt, when it is 2 pm in Mexico City?”). Answers were again compared to a predefined solution.

As *background music*, we used two different common German songs: “Auf uns” by *Andreas Bourani* and “Nur ein Wort” by *Wir sind Helden*, both in the instrumental version. Both songs were chosen to induce positive mood. According to Thompson et al.’s (2011) results, we chose two songs with a fast tempo and presented them at a medium volume (30%) to not disturb the participants too much. The songs were presented through over-ear headphones. The two songs were played between the recorded instructions to start and stop reading. To not induce any motivational effects, participants in the control group also wore headphones but only heard the instructions to start and stop reading.

*Working memory capacity* was measured with the computer-based Numerical Memory Updating Test (Oberauer et al., 2000). Digits that are shown in a spatial matrix for seconds have to be stored and processed by simple additions and subtractions. The resultant capacity scores indicate how many of the nine matrix fields learners can process simultaneously.

*Arousal* was measured before and after learning with the subscale of the Self-Assessment Manikin (Bradley and Lang, 1994). This questionnaire measures arousal with a 9-point Likert-Scale ranging from 1 = “highly aroused” to 9 = “not at all aroused,” which is illustrated by a pictorial representation of a stick figure with more or less arousal indicated by a bigger or smaller explosion in its belly.

To measure *mood* before and after learning, we used a short version of the Multidimensional Mood State Questionnaire (Steyer et al., 2004). The questionnaire consisted of 14 emotions grouped into 3 subscales: good-bad-mood (angry, happy, joyful, satisfied, unhappy, and well), awake-tired (awake, lively, rested, and tired), and calm-nervous (balanced, nervous, relaxed, and restless). Participants scored each emotion according to the question “Please score how you feel at the moment.” The answer format was a 7-point Likert-Scale ranging from 1 = “completely true” to 7 = “not true.” A positive score in a subscale denotes positive emotions (being in a good mood, awake, and calm), a negative score indicates negative emotions (being in a bad mood, tired, and nervous). To calculate the influence of the learning phase on emotions, we subtracted mood values before learning from values after learning. Thus, a positive value in our study symbolizes an increase in positive emotions (good mood, awake, and calm) whilst a negative value indicates an increase in negative emotions (bad mood, tired, and nervous).

In addition, we used a *demographic questionnaire* to assess each learner’s age, gender and study subject. The questionnaire also included questions concerning the musical expertise of our participants: Did they have experience of singing in a choir and if so, for how many years? Did they have experience playing an instrument and if so, for how many years? Moreover, we asked participants to score how musical they would assess themselves to be on a 7-point Likert-scale. Furthermore, after the learning phase, we asked the participants in the condition with background music if they were familiar with the song they had listened to.

### Procedure

Data collection took place in group sessions. First, participants were asked to formally agree to participate in the experiment and the involved data collection by signing the informed consent form. This informed the participants about the duration and tasks involved in the experiment, that data will be used anonymously, the possibility to ask questions during the data collection and to withdraw their participation at any time. All participants who agreed to the data collection then completed the demographic questionnaire, two pre-tests for arousal and mood as well as a test of prior knowledge. Following this, the learning phase took place: Participants were asked to put on the headphones and to start their track, consisting of either the instructions to start and stop learning or the same instructions but with the two songs played in between. After the learning phase, participants completed the arousal and mood questionnaires again. The post-test then took place. The whole data collection took approximately 45 min.

### Covariates

To identify potential covariates, we checked whether prior knowledge, age and gender were equally distributed between the conditions. As we did not find any significant differences (all *p*s > 0.35), we did not include any covariates in further analyses.

Moreover, we analyzed whether musical experience (experience singing or playing an instrument) or familiarity with the songs influenced recall or comprehension. We did not find any significant differences between the groups (all *p*s > 0.35). Thus, musical experience and familiarity with the songs were not considered further.

## Results

### Descriptive Data

Descriptive data concerning all dependent variables in all conditions can be found in **Table 1**.

**Table 1:** Descriptive data for all variables per condition.

	Conditions															
	*Presence of background music*															
	Without background music								With background music							
	*Working memory capacity*															
	2 (*n* = 5)		3 (*n* = 16)		4 (*n* = 10)		5 (*n* = 8)		2 (*n* = 6)		3 (*n* = 15)		4 (*n* = 7)		5 (*n* = 14)	
	*M*	*SD*	*M*	*SD*	*M*	*SD*	*M*	*SD*	*M*	*SD*	*M*	*SD*	*M*	*SD*	*M*	*SD*
Prior knowledge (%)	45.00	17.28	22.92	15.67	35.00	19.95	32.29	19.64	8.33	7.45	28.33	21.32	26.19	17.63	33.93	15.49
Recall (%)	44.00	5.48	33.13	17.01	43.00	17.67	50.00	9.26	31.37	9.83	42.67	15.34	41.43	10.69	38.57	21.07
Comprehension (%)	70.00	14.14	48.75	22.47	57.00	24.97	72.50	14.88	33.33	13.66	58.67	24.16	55.71	27.60	65.00	19.12
Arousal (Difference after-before)^∗^	-0.20	0.84	-0.81	1.60	-0.89	0.78	0.13	0.99	-0.33	1.37	-0.77	1.69	0.14	1.07	-0.69	2.02
Good-Bad-Mood (Difference after-before)^∗^	0.60	4.67	0.50	5.40	-1.70	5.88	1.38	3.50	-1.83	4.79	-0.64	6.39	1.14	5.01	1.21	6.68
Awake-Tired (Difference after-before)^∗^	-0.80	1.64	-0.06	3.68	-1.60	2.51	-0.44	3.84	1.33	3.14	-0.86	4.09	-0.29	2.56	0.71	4.99
Calm-Nervous (Difference after-before)^∗^	-1.00	4.64	0.75	3.57	-1.00	3.37	-0.38	3.84	1.17	5.04	-0.69	4.33	1.43	5.65	1.00	3.37

*^∗^ Positive values indicate an increase in this scale, negative values a decrease.*

### Potential Mediators

To analyze whether background music influences learning outcomes indirectly mediated through mood or arousal, a first step is to analyze whether background music influences mood or arousal directly. If so, we will then analyze whether these variables influence learning outcomes significantly (for a theoretical approach concerning mediator analyses, see Baron and Kenny, 1986).

#### Arousal

Listening to background music did not influence the difference in arousal before and after learning, *F* < 1, ns. The prerequisites for a mediation were not reached in this case.

#### Mood

Background music did not influence the differences in moods before and after learning in the good-bad mood subscale or in the awake-tired subscale, *Fs* < 1, ns, nor the calm-nervous subscale, *F*(1,77) = 1.04, ns, η^2^ = 0.01. Again, the prerequisites for a mediation were not reached.

### Recall

Neither the presence of background music, *F*(3,73) = 1.08, ns, η^2^ = 0.02, nor working memory capacity, *F* < 1, ns, or the interaction between both factors, *F*(3,73) = 2.37, ns, η^2^ = 0.09, influenced recall significantly.

### Comprehension

The presence or absence of background music, *F*(1,73) = 2.90, *p* = 0.046, η^2^ = 0.04, influenced comprehension outcomes with no background music leading to better comprehension. Moreover, working memory capacity affected comprehension, *F*(3,73) = 2.44, *p* = 0.035, η^2^ = 0.09, with learners with high capacity reaching better comprehension scores. A planned *post hoc* contrast revealed higher comprehension scores for participants with a working memory score of 5 than participants with a working memory score of 2 (*MD* = 17.73, *SE* = 8.23, *p* = 0.017, *d* = 0.86) or 3 (*MD* = 14.18, *SE* = 6.25, *p* = 0.013, *d* = 0.68). All other contrasts failed to show significant results.

**FIGURE 1:**
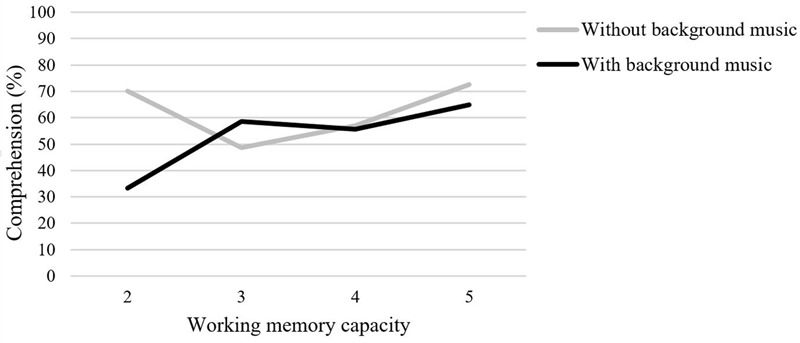
Interaction between background music and working memory capacity on comprehension.

The interaction between background music and working memory capacity was significant, *F*(3,73) = 3.22, *p* < 0.028, η^2^ = 0.12 (see **Figure 1**). Planned *post hoc* contrast compared comprehension scores within the same working memory score and between the experimental groups. We found higher comprehension scores for participants with the lowest working memory score of 2 in the group with no music compared to the group with music (*MD* = 36.67, *SE* = 13.06, *p* = 0.003, *d* = 2.64). There were no significant differences in any other contrast. Analyzing both experimental groups separately, it appears that the results of the group without background music follow a quadratic trend (*MD* = 18.38, *SE* = 7.36, *p* = 0.017), while the results of the group with background music follow a linear trend (*MD* = 20.58, *SE* = 7.55, *p* = 0.010).

## Discussion

The aim of this study was firstly, to examine whether background music has a direct effect on learning outcomes or whether this influence is mediated by arousal and mood. Secondly, we wanted to investigate whether the influence background music has on learning outcomes could be positive, for instance when listening to a song with specific facilitative characteristics, or whether, following the seductive detail assumption, a cognitive burden would always be present. Finally, we wanted to examine which role the learner’s working memory capacity or its interaction with background music has in, speaking about learning outcomes. Results will be discussed referring to these research questions.

### Mediation Effect or Direct Influence of Background Music?

To investigate whether there is a mediation effect of background music through arousal and mood on learning, we first calculated differences in arousal and mood before and after learning. As a second step, we tested whether these scores were different between the groups with or without background music during the learning phase. As there were no significant differences between the conditions, we inferred that in this study background music did not affect arousal or mood. This is contradictory to the results of previous studies (e.g., Nantais and Schellenberg, 1999; Juslin and O’Neill, 2001; Sloboda and Juslin, 2001; Husain et al., 2002; Pelletier, 2004; Schmidt and Trainor, 2010). We provide three possible explanations for these contradictory results: Firstly, the time span during which the participants were exposed to the music might have been too short to have had an impact. Learning phases in everyday life are usually much longer than in our experiment and learners may normally be exposed to music for longer periods. It might be the case, that it is necessary to listen to music for a longer time period to affect arousal or mood.

Secondly, the measurement tool might not have been sensitive enough to measure small changes in mood or arousal. The Likert scales used in this experiment consisted of seven and nine gradations of mood and arousal, respectively. Thus, in between two adjacent scale responses (e.g., between a 4 or 5) there is a 14% differences in variance in the mood scale and 11% in the arousal scale. If the influence of listening to background music was smaller than this, the measurement tool would simply not be able to account for the differences. A possible alternative approach would be to use a continuous scale. In addition, arousal could also be measured objectively with physiological data, such as heart rate, blood pressure or skin conductance.

Thirdly, contradictory to both recent explanations, it might be the case that the specific background music we used simply does not influence arousal or mood in a learning scenario such as ours. The two songs were picked based on the results of earlier studies concerning song characteristics. We chose fast paced songs to induce arousal and played them at a medium volume in line with Thompson et al.’s (2011) findings. Moreover, we used songs with a positive sounding melody which have positive lyrics in their original version. Nevertheless, it could be the case that these characteristics did not fit our sample in terms of music taste. For example, if a section of our sample did enjoy the music genre whilst the others did not the positive and negative effects may cancel each other out. This idea is supported by the rather high standard deviations in the scales, as well as the different high scores between the different levels of working memory capacity, see **Table 1**. Moreover, contradictory to Thompson et al.’s (2011) findings Hallam et al. (2002, study 2) found that fast music negatively influenced learning outcomes. This contradiction emphasizes how important it is to control for learners’ characteristics in studies and, in addition, to be precise with the description of the musical stimuli, so that “fast music” is understood in replicable terms in all studies.

In summary, we were not able to confirm the arousal-mood-hypothesis, as background music did not affect arousal or mood in our study. However, besides arousal and mood, there are other learners’ characteristics which could potentially be mediators not tested in this study, such as learner motivation. Anyway, did background music have a direct, positive or negative influence on learning outcomes in this study?

Concerning recall, background music did not influence performance, confirming our hypothesis. Therefore, the potential positive effect on cognitive abilities postulated by Rauscher et al. (1993) and the seductive detail effect (Rey, 2012) either do not benefit the learner or indeed cancel each other out. As recall tasks only place as small burden on working memory, there is still enough capacity left after processing background music. A study by Brünken et al. (2004) supports this idea as they did not find an influence of listening to background music on cognitive load while completing a simple recall task. Thus, background music did not influence recall negatively. We believe that there is neither a positive, nor a negative impact on recall and no compensation effect. However, if one would like to affect recall through music, some success has been found by using jingles to improve recall for short verbal sequences (e.g., Yalch, 1991; VanVoorhis, 2002).

When considering comprehension, learners reached higher levels of learning with no background music. This result lends support to our seductive detail hypothesis (1.2c): As background music is always processed first (Salamé and Baddeley, 1989) there is not enough capacity left to work on cognitively demanding comprehension tasks. In conclusion, this was the only association which we found between background music and learning outcomes, direct or indirect. This indicates that besides the arousal-mood-hypothesis, the Mozart effect hypothesis also needs to be rejected. In this study, background music functioned as a seductive detail for more demanding learning processes such as comprehension.

A further point which needs to be considered is that the songs we used were instrumental versions of popular songs with lyrics. Even though we did not present the lyrics they may have been activated by the melody as an anchor (see for example, Bartlett and Snelus, 1980; Wallace, 1994). On the one hand, the activated lyrics interfere with the text the participants have to learn in working memory, as participants would have to deal with both simultaneously. On the other hand, participants would need less effort to process the melody, as familiar information is easier to process than unfamiliar information (Hulme et al., 1991). Taken together, the negative and positive effects may cancel each other out and may explain why in our study, we did not find any influence of learners’ familiarity with the songs on learning outcomes.

### Working Memory Capacity

Answering our second research question, working memory capacity did not influence recall performance As in the explanation above, recall tasks do not demand much cognitive capacity and because of this, all learners should be able to process the relevant content, independent of their working memory capacity. However, comprehension tasks require more cognitive capacity. Hence, in support of our hypothesis, learners with higher working memory capacity reached higher comprehension scores as they are able to process more units of information simultaneously allowing them to better understand the test.

### Interaction between Background Music and Working Memory Capacity

The last research question concerned the interaction between background music and learners’ working memory capacities. In the case of the recall tasks, neither background music nor working memory capacity played a crucial role. Even learners with little capacity should be able to process background music in addition. Indeed, we found conformation of our hypothesis that the interaction between both factors did not influence recall performance.

In the case of comprehension, however, we found a significant interaction between listening to background music and working memory capacity. The only significant and relevant contrast occurred in the learners with the lowest working memory capacity who reached higher comprehension scores without background music. As their working memory capacity is highly limited, they are simply not able to process a comprehension tasks and background music simultaneously. For all of the other capacity levels we did not find such a difference or indeed, any advantages when learning with music. This finding is also in keeping with the seductive detail assumption and comparable to the ability-as-compensator effect (Mayer and Sims, 1994).

In line with this result, we found a linear trend in the group which learned with background music. The higher a learner’s working memory capacity, the better they learn with background music. Whilst processing the music, they still have enough capacity left for the main learning task. We found a quadratic trend when analyzing the group without background music. As expected, learners with medium working memory capacity performed worse than those with high working memory capacity scores. Unexpectedly, learners with low working memory capacity scores outperformed the medium capacity groups and their results matched that of the high-capacity group. We expected a better performance with increasing capacity. However, Zander (2010) found that some learners may not constantly invest all of their capacities in the learning process, so that learners with beneficial learning characteristics do not necessarily outperform those learners with poor skills. In this context we also need to point out that our sample for the extreme group analysis was rather small. Therefore, effects might also have been attributed to other variables such as motivation or situational interest, which might be unequally distributed and were not controlled for.

### Limitations and Further Research

As in all studies involving music, these results are not simply transferable to learning with other songs. If at all, one would expect similar results when using songs with the same characteristics, such as tempo or mode. The background music in this study did not influence arousal or mood as expected. It is therefore important that a learner’s attitude concerning the presented music need to be taken into account. Further research need to investigate whether one would reach the same results while testing participants with different characteristics. Furthermore, the direct negative influence of background music needs further investigation. Even though we found evidence of a seductive detail effect, this result needs to be validated by measuring cognitive load after learning with and without background music, and differentiated for all three types of load during solving recall and comprehension tasks. For this, one could use the cognitive load questionnaire developed by Leppink et al. (2013). Furthermore, it would be interesting to assess how exactly background music impacts learning on a cognitive basis: For example, the question of how exactly background music is processed is still an open one.

Moreover, as mentioned above, we recommend using a more sensitive measuring tool than we did. Our tools were not able to detect small variations in either arousal or mood. We would suggest using continual instruments to pick up on subtle chances in variance.

In addition, working memory capacity is also discussed as being relevant in the context of creativity (e.g., Jalil, 2007; Vandervert et al., 2007; Sharma and Babu, 2017). Therefore, it might be interesting for further research to consider creativity as another aptitude variable in the context of learning with background music. For example, we could imagine that highly creative learners may especially benefit from listening to background music while learning. Moreover, it could also be relevant to measure the impact of the interaction between background music and working memory capacity on creative learning tasks.

### Practical Implications

Based on the results of this study, we cannot recommend learning with background music. Learners with the lowest capacity levels were especially impaired by background music. With increasing working memory capacity background music neither hindered nor fostered learning. For these learners it is merely a matter of personal preference as to whether they wish to learn with background music or not, for example in an attempt to raise their motivation levels. However, learners should be careful with their decision as to which music they chose to listen to: Song with lyrics are potentially more distracting than instrumental melodies and music with other modes or tempos could possibly evoke obstructive emotions for learning. Luckily, there is enough music readily available, so that each of us has the chance to listen to our preferred music, which may even be conducive to learning.

## Ethics Statement

Our study is about learning with or without background music. There was no potential to harm or endanger any participants. Moreover, we did not collect any sensitive data. Hence, there was no offical ethics approval needed.

## Author Contributions

JL designed and conducted this study and wrote this manuscript – all under supervision of TS.

## Conflict of Interest Statement

The authors declare that the research was conducted in the absence of any commercial or financial relationships that could be construed as a potential conflict of interest.

## References

[B1] Alloy T. P. Alloy R. G. (2010). Investigating the predictive roles of working memory and IQ in academic attainment. *J. Exp. Child Psychol.* 106 20–29. 10.1016/j.jecp.2009.11.003 20018296

[B2] Baddeley A. (1986). *Working Memory.* Oxford: Oxford University Press.

[B3] Barnett V. Lewis T. (1994). *Outliers in Statistical Data.* Hoboken: John Wiley & Sons.

[B4] Baron R. M. Kenny D. A. (1986). The moderator-mediator variable distinction in social psychological research: conceptual, strategic, and statistical considerations. *J. Pers. Soc. Psychol.* 6 1173–1182. 10.1037/0022-3514.51.6.1173 3806354

[B5] Bartlett J. C. Snelus P. (1980). Lifespan memory for popular songs. *Am. J. Psychol.* 93 551–560. 10.2307/1422730

[B6] Bloom B. (1956). *Taxonomy of Educational Objectives: The Classification of Educational Goals (Handbook I: Cognitive domain).* New York, NY: McKay.

[B7] Bradley M. M. Lang P. J. (1994). Measuring emotion: the self-assessment manikin and the semantic differential. *J. Behav. Ther. Exp. Psychiatry* 25 49–59. 10.1016/0005-7916(94)90063-9 7962581

[B8] Brünken R. Plass J. L. Leutner D. (2004). Assessment of cognitive load in multimedia learning with dual-task methodology: auditory load and modality effects. *Instr. Sci.* 32 115–132. 10.1023/B:TRUC.0000021812.96911.c5

[B9] Cowan N. (1999). “An embedded-processes model of working memory,” in *Models of Working Memory: Mechanisms of Active Maintenance and Executive Control* eds Miyake E. Shah P. (Cambridge: Cambridge University) 62–101.

[B10] Cowan N. (2001). The magical number 4 in short-term memory: a reconsideration of mental storage capacity. *Behav. Brain Sci.* 24 87–185. 10.1017/S0140525X01003922 11515286

[B11] Cowan N. (2012). *Working Memory Capacity.* Abingdon: Psychology Press.

[B12] Daneman M. Carpenter P. A. (1983). Individual differences in integrating information between and within sentences. *J. Exp. Psychol.* 9 561–583. 10.1007/s11571-014-9292-2 26396645 PMC4571645

[B13] de Groot A. M. B. (2006). Effects of stimulus characteristics and background music on foreign language vocabulary learning and forgetting. *Lang. Learn.* 56 463–506. 10.1111/j.1467-9922.2006.00374.x

[B14] Deutsch D. (1970). Tones and numbers: specificity of interference in immediate memory. *Science* 168 1604–1605. 10.1126/science.168.3939.1604 5420547

[B15] Eysenck M. W. (1976). Arousal, learning and memory. *Psychol. Bull.* 83 389–404. 10.1037/0033-2909.83.3.389 778883

[B16] Fenesi B. Kramer E. Kim J. A. (2016). Split-attention and coherence principles in multimedia instruction can rescue performance for learners with lower working memory capacity. *Appl. Cogn. Psychol.* 30 691–699. 10.1002/acp.3244

[B17] Foss D. J. (1982). A discourse on semantic priming. *Cogn. Psychol.* 14 590–607. 10.1016/0010-0285(82)90020-2 7140212

[B18] Furnham A. Bradley A. (1997). Music while you work: the differential distraction of background music on the cognitive test performance of introverts and extroverts. *Appl. Cogn. Psychol.* 11 445–455. 10.1002/(SICI)1099-0720(199710)11:5<445::AID-ACP472>3.0.CO;2-R

[B19] Goetz T. Hall N. C. (2013). “Emotion and achievement in the classroom,” in *International Guide to Student Achievement* eds Hattie J. Anderman E. M. (Abingdon: Routledge) 192–195.

[B20] Hallam S. Price J. Katsarou G. (2002). The effect of background music on primary school pupils’ task performance. *Educ. Stud.* 28 111–122. 10.1080/03055690220124551

[B21] Heuer F. Reisberg D. (2014). “Emotion, arousal and memory for detail,” in *The Handbook of Emotion and Memory: Research and Theory* ed. Christianson S.-A. (New York, NY: Psychology Press).

[B22] Hulme C. Maughan S. Brown G. D. A. (1991). Memory for familiar and unfamiliar words: Evidence for a long-term memory contribution to short-term memory. *J. Mem. Lang.* 30 685–701. 10.1016/0749-596X(91)90032-F

[B23] Husain G. Thompson W. F. Schellenberg E. G. (2002). Effects of musical tempo and mode on arousal, mood and spatial abilities. *Music Percept.* 20 151–171. 10.1525/mp.2002.20.2.151

[B24] Ilsen A. M. (1984). “Towards understanding the role of affect in cognition,” in *Handbook of Social Cognition* eds Wyer R. Srull T. (Hillsdale, NJ: Lawrence Erlbaum Associates) 179–236.

[B25] Isen A. M. (2002). An influence of positive affect on decision making in complex situations: theoretical issues with practical implications. *J. Consum. Psychol.* 11 75–85. 10.1207/S15327663JCP1102_01

[B26] Jalil P. A. (2007). Working memory, cerebellum, and creativity. *Creat. Res. J.* 19 39–45. 10.1080/10400410709336878

[B27] Jäncke L. Sandmann P. (2010). Music listening while you learn: no influence of background music on verbal learning. *Behav. Brain Funct.* 6 1–14. 10.1186/1744-9081-6-3 20180945 PMC2828975

[B28] Juslin P. N. O’Neill S. A. (2001). “Psychological perspectives on music and emotion,” in *Music and Emotion: Theory and Research* eds Juslin P. N. Sloboda J. A. (New York, NY: Oxford University) 71–104.

[B29] Kämpfe J. Sedlmeier P. Renkewitz F. (2010). The impact of background music on adult listeners: a meta-analysis. *Psychol. Music* 39 424–448. 10.1177/0305735610376261

[B30] King J. Just M. A. (1991). Individual differences in syntactic processing: the role of working memory. *J. Mem. Lang.* 30 580–602. 10.1016/0749-596X(91)90027-H

[B31] Kleinsmith L. J. Kaplan S. (1963). Paired-associate learning as a function of arousal and interpolated interval. *J. Exp. Psychol.* 65 190–193. 10.1037/h0040288 14033436

[B32] Lehmann J. Goussios C. Seufert T. (2016). Working memory capacity and disfluency effect: an aptitude-treatment-interaction study. *Metacogn. Learn.* 11 89–105. 10.1007/s11409-015-9149-z

[B33] Leppink J. Paas F. Van Der Vleuten C. Van Gog T. Van Merrienboer J. (2013). Development of an instrument for measuring different types of cognitive load. *Behav. Res. Methods* 45 1058–1072. 10.3758/s13428-013-0334-1 23572251

[B34] Mayer R. E. (2001). *Multimedia Learning.* New York, NY: Cambridge University Press.

[B35] Mayer R. E. Sims V. K. (1994). For whom is a picture worth a thousand words? Extensions of a dual-coding theory of multimedia learning. *J. Educ. Psychol.* 86 389–401. 10.1037/0022-0663.86.3.389

[B36] Miller G. A. (1994). The magical number seven, plus or minus two: some limits on our capacity for processing information. *Psychol. Rev.* 101 343–352. 10.1037/0033-295X.101.2.343 8022966

[B37] Moreno R. Mayer R. (2000). A coherence effect in multimedia learning: the case for minimizing irrelevant sounds in the design of multimedia instructional messages. *J. Educ. Psychol.* 92 117–125. 10.1037/0022-0663.92.1.117

[B38] Nantais K. M. Schellenberg E. G. (1999). The mozart effect: an artifact of preference. *Psychol. Sci.* 10 370–373. 10.1111/1467-9280.00170

[B39] Oberauer K. Süß H.-M. Schulze R. Wilhelm O. Wittmann W. W. (2000). Working memory capacity— facets of a cognitive ability construct. *Pers. Individ. Diff.* 29 1017–1045. 10.1016/S0191-8869(99)00251-2

[B40] O’Hanlon J. F. (1981). Boredom: practical consequences and a theory. *Acta Psychol.* 49 53–82. 10.1016/0001-6918(81)90033-0 7304249

[B41] Paivio A. Phlipchalk R. Rowe E. J. (1975). Free and serial recall of pictures, sounds, and words. *Mem. Cogn.* 3 586–590. 10.3758/BF03198221 24203897

[B42] Park B. Moreno R. Seufert T. Brünken R. (2011). Does cognitive load moderate the seductive details effect? A multimedia study. *Comput. Hum. Behav.* 27 5–10. 10.1016/j.chb.2010.05.006

[B43] Pekrun R. (2006). The control-value theory of achievement emotions: assumptions, corollaries, and implications for educational research and practice. *Educ. Psychol. Rev.* 18 315–341. 10.3109/0142159X.2012.643265 22364457

[B44] Pekrun R. Lichtenfeld S. Marsh H. W. Murayama K. Goetz T. (2017). Achievement emotions and academic performance: longitudinal models of reciprocal effects. *Child Dev.* 88 1653–1670. 10.1111/cdev.12704 28176309

[B45] Pelletier C. L. (2004). The effect of music on decreasing arousal due to stress: a meta-analysis. *J. Music Ther.* 3 192–214. 10.1093/jmt/41.3.192 15327345

[B46] Perham N. Currie H. (2014). Does listening to preferred music improve reading comprehension performance? *Appl. Cogn. Psychol.* 28 279–284. 10.1002/acp.2994

[B47] Randsell S. E. Gilroy L. (2001). The effects of background music on word processed writing. *Comput. Hum. Behav.* 17 141–148. 10.1016/S0747-5632(00)00043-1

[B48] Rauscher F. H. Shaw G. L. Ky K. N. (1993). Music and spatial task performance. *Nature* 365 611. 10.1038/365611a0 8413624

[B49] Rey G. (2012). A review of research and a meta-analysis of the seductive detail effect. *Educ. Res. Rev.* 7 216–237. 10.1016/j.edurev.2012.05.003

[B50] Rosen V. M. Engle R. W. (1998). Working memory capacity and suppression. *J. Mem. Lang.* 39 418–436. 10.1006/jmla.1998.2590

[B51] Rowe E. Philipchalk R. Cake L. (1974). Short-term memory for sounds and words. *J. Exp. Psychol.* 102 1140–1142. 10.1037/h0036379

[B52] Rowe E. J. (2013). Ordered recall of sounds and words in short-term memory. *Bull. Psychon. Soc.* 4 559–561. 10.3758/BF03334290

[B53] Salamé P. Baddeley A. D. (1989). Effects of background music on phonological short-term memory. *Q. J. Exp. Psychol.* 41 107–122. 10.1080/14640748908402355 9206583

[B54] Sanchez C. A. Wiley J. (2006). An examination of the seductive details effect in terms of working memory capacity. *Mem. Cognit.* 34 344–355. 10.3758/BF03193412 16752598

[B55] Savan A. (1999). The effect of background music on learning. *Psychol. Music Music Educ.* 27 138–146. 10.1177/0305735699272005

[B56] Scheree A. Henke J. McLaughlin M. Ripp M. Tuffs P. (2000). Using background music to enhance memory and improve learning. *Paper Presented at the 24th Annual OCD Conference* San Francisco, CA.

[B57] Schmidt L. A. Trainor L. J. (2010). Frontal brain electrical activity (EEG) distinguishes valence and intensity of musical emotions. *Cogn. Emot.* 15 487–500. 10.1080/02699930126048

[B58] Schnotz W. Bannert M. (1999). Einflüsse der Visualisierungsform auf die Konstruktion mentaler Modelle beim Text- und Bildverstehen [Influence of the type of visualization on the construction of mental models during picture and text comprehension]. *Z. Exp. Psychol.* 46 217–236. 10.1026//0949-3964.46.3.217 10474324

[B59] Schwartz R. W. Ayres K. M. Douglas K. H. (2017). Effect of music on task performance, engagement, and behavior: a literature review. *Psychol. Music.* 45 611–627. 10.1177/0305735617691118

[B60] Sharma S. Babu N. (2017). Interplay between creativity, executive function and working memory in middle-aged and older adults. *Creat. Res. J.* 29 71–77. 10.1080/10400419.2017.1263512

[B61] Sloboda J. A. Juslin P. N. (2001). “Psychological perspectives on music and emotion,” in *Music and Emotion: Theory and Research* eds Juslin P. N. Sloboda J. A. (New York, NY: Oxford University) 71–104.

[B62] Steyer R. Schwenkmezger P. Notz P. Eid M. (2004). *Entwicklung des Mehrdimensionalen Befindlichkeitsfragebogens (MDBF). Primärdatensatz [Development of the Multidimensional Mood State Questionnaire (MDBF). Primary Data.].* Trier: Center for Research Data in Psychology.

[B63] Sweller J. (2010). “Cognitive load theory: recent theoretical advances,” in *Cognitive Load Theory* eds Plass J. L. Moreno R. Brünken R. (New York, NY: Cambridge University Press) 29–47.

[B64] Sweller J. Ayres P. Kalyuga S. (2011). *Cognitive Load Theory.* New York, NY: Springer.

[B65] Thompson W. F. Schellenberg E. G. Letnic A. K. (2011). Fast and loud background music disrupts reading comprehension. *Psychol. Music* 40 700–708. 10.1177/0305735611400173

[B66] Vandervert L. R. Schimpf P. H. Liu H. (2007). How working memory and the cerebellum collaborate to produce creativity and innovation. *Creat. Res. J.* 19 1–18. 10.1080/10400410709336873

[B67] VanVoorhis C. R. W. (2002). Stat jingles: to sing or not to sing. *Teach. Psychol.* 29 249–250.

[B68] Wallace W. (1994). Memory for music: effect of melody on recall of text. *J. Exp. Psychol.* 20 1417–1485. 10.1037/0278-7393.20.6.1471

[B69] Whitney P. Ritchie B. G. Clark M. B. (1991). Working-memory capacity and the use of elaborative inferences in text comprehension. *Discourse Process.* 14 133–145. 10.1080/01638539109544779

[B70] Yalch R. F. (1991). Memory in a jingle jungle: music as a mnemonic device in communicating advertising slogans. *J. Appl. Psychol.* 76 268–275. 10.1037/0021-9010.76.2.268

[B71] Yerkes R. M. Dodson J. D. (1908). The relation of strength of stimulus to rapidity of habit-formation. *J. Comp. Neurol. Psychol.* 18 459–482. 10.1002/cne.920180503

[B72] Zander S. (2010). *Motivationale Lernervoraussetzungen in der Cognitive Load Theory. Zwei Studien zum Einfluss motivationaler Lernervoraussetzungen auf die Kognitive Belastung beim Lernen mit Unterschiedlichen Instruktionsdesigns [Motivational Learner’s Characteristics and Cognitive Load Theory. Two Studies About the Impact of Motivational Learner’s Characteristics on Cognitive Load While Learning with Different Instructional Designs].* Berlin: Logos Verlag Berlin GmbH.

